# Hystérocèle géante

**DOI:** 10.11604/pamj.2020.37.33.20947

**Published:** 2020-09-08

**Authors:** Ahmed Ibrahimi, Idriss Ziani

**Affiliations:** 1 Service d´Urologie-A, Centre Hospitalo-Universitaire Ibn Sina, Faculté de Médecine et de Pharmacie, Université Mohammed V, 10000, Rabat, Maroc

**Keywords:** Prolapsus génito-urinaire, hystérocèle, hystéroptose, hystérectomie, Genitourinary prolapse, hysterocele, hysteroptosis, hysterectomy

## Abstract

The genitourinary prolapse is a common disorder in women. It may affects three pelvic organs. The hysterocele or hysteroptosis is defined as the descent of the uterus into vagina and, in severe cases, it protrudes out of the vulva. It is more often secondary to a lack of support and suspension of the pelvic organs. We report the case of a 58-year-old woman, with no particular past medical history, who reached menopause six years before and had had two vaginal deliveries. She presented with dysuria, pollakiuria, recurrent urinary tract infections and recurrent episodes of acute retention of urine, with a sensation of pelvic heaviness and vaginal ball externalizing due to effort and at the end of the day. The clinical examination highlighted a huge stage IV hysterocele according to Baden Walker classification, with complete cervix externalization, without urinary incontinence, cystocele or associated rectocele. The patient underwent vaginal hysterectomy with good functional outcome, and disappearance of associated urinary disorders.

## Image en médecine

Le prolapsus génito-urinaire est une pathologie fréquente de la femme, il peut intéresser les organes des trois compartiments pelviens. L’hystérocèle ou l'hystéroptose se définit comme la descente de l'utérus à travers le vagin et dans les cas sévères son extériorisation à travers la vulve, elle est le plus souvent secondaire à un défaut de soutènement et de suspension des organes pelviens. Nous rapportons le cas d'une patiente âgée de 58 ans, sans antécédents pathologiques particuliers, ménopausée depuis six ans, avec deux accouchements par voie basse qui consulte pour dysurie, pollakiurie, infection urinaire à répétition et des épisodes récurrents de rétention aiguë des urines, avec sensation de pesanteur pelvienne et de boule intravaginale s'extériorisant à l'effort et en fin de journée. L'examen clinique a mis en évidence une énorme hystérocèle stade IV selon la classification de Baden Walker, avec un col utérin complètement extériorisé sans incontinence urinaire, cystocèle ou de rectocèle associé. La patiente a bénéficié d'une hystérectomie par voie basse avec un bon résultat fonctionnel, et disparition des troubles urinaires associés.

**Figure 1:**
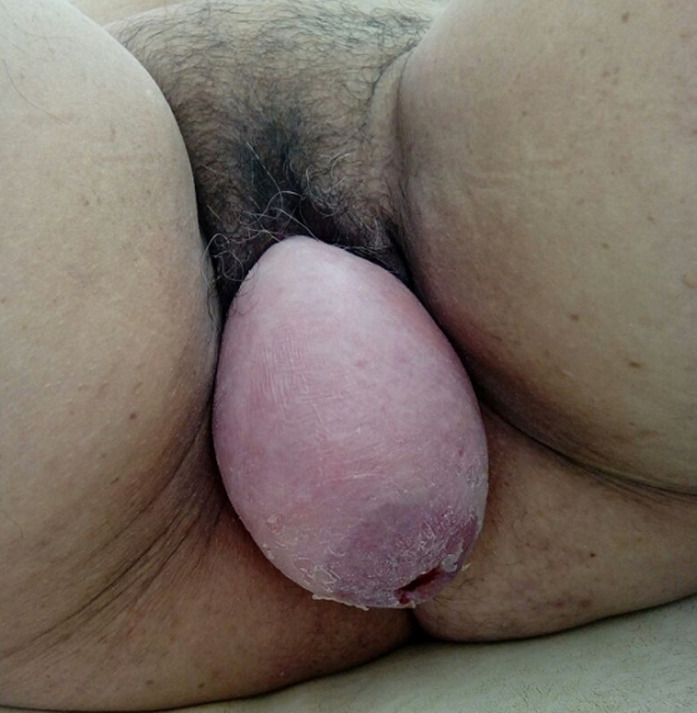
énorme hystérocèle stade IV avec éversion complète de l´utérus à travers la vulve

